# Exploring the Pathophysiology of Reinke's Edema: The Cellular Impact of Cigarette Smoke and Vibration

**DOI:** 10.1002/lary.28855

**Published:** 2020-06-22

**Authors:** Tanja Grossmann, Barbara Steffan, Andrijana Kirsch, Magdalena Grill, Claus Gerstenberger, Markus Gugatschka

**Affiliations:** ^1^ Division of Phoniatrics Medical University of Graz Graz Austria

**Keywords:** Reinke's edema, vocal fold fibroblasts, cigarette smoke, benign vocal fold lesions, phonomimetic bioreactor

## Abstract

**Objectives:**

To explore the isolated or combined effects of cigarette smoke extract (CSE) and vibration on human vocal fold fibroblasts (hVFF) in an in vitro setting in order to elucidate their influence in the pathophysiology of Reinke's edema (RE).

**Study design:**

Immortalized hVFF were exposed to CSE or control medium under static or vibrational conditions. A phonomimetic bioreactor was used to deliver vibrational patterns to hVFF over a period of 5 days.

**Methods:**

Cytotoxicity was quantified using a lactate dehydrogenase assay. We employed reverse transcription–quantitative polymerase chain reaction, enzyme‐linked immunosorbent assay, and Magnetic Luminex^(R)^ assays (R&D Systems, Minneapolis, MN) to assess the influence on extracellular matrix production, fibrogenesis, inflammation, and angiogenesis.

**Results:**

We observed significant changes induced by CSE alone (hyaluronic acid, matrix metalloproteinase 1, Interleukin‐8, cyclooxygenase [COX]1, COX2, vascular endothelial growth factor [VEGF]D), as well as settings in which only the combination of CSE and vibration led to significant changes (transforming growth factor beta 1, VEGFA, VEGFC). Also, CSE‐induced levels of COX2 were only significantly reduced when vibration was applied.

**Conclusion:**

We were able to explore the cellular effects of CSE and vibration on hVFF by employing a phonomimetic bioreactor. Whereas cigarette smoke is generally accepted as a risk factor for RE, the role of vibration remained unclear as it is difficult to study in humans. Our data showed that some genes and proteins in the pathophysiological context of RE were only affected when CSE in combination with vibration was applied.

**Level of Evidence:**

NA *Laryngoscope*, 131:E547–E554, 2021

## INTRODUCTION

Reinke's edema (RE) is a benign, bilaterally occurring polypoid lesion of the vocal folds (VF) leading to dysphonia and a lowered voice. The most relevant and generally accepted risk factor is smoking.^1^, ^2^ Likewise, vocal abuse and laryngo‐pharyngeal reflux (LPR) are often regarded as additional risk factors, albeit their separate impact remains unclear. Furthermore, a predominance in elderly females is reported, suggesting a contribution of the hormonal status.^3^, ^4^, ^5^ Therapeutic recommendations are based on a cessation of these risk factors (smoking), a probatory treatment of LPR, and ultimately phonosurgery. The latter leads mostly to an improvement of dysphonia; however, it cannot restore the healthy state. In more than 80% of the cases, histological features of RE comprise epithelial hyperplasia, thickening of the basement membrane, inflammation, and fibrosis.^6^, ^7^ Prolongated and dilated vessels are a prominent feature in typical RE, which can be seen during laryngoscopy.^8^ Electron microscopical examinations showed that these vessels were typically fenestrated with an increased permeability leading to edema.^9^ However, edema of the lamina propria already occurs in the early stages of smoking abuse. By using a large smoking chamber, Liu et al. proved the development of a significant edema in a porcine model after exposure to 15 cigarettes per day for 20 days.^10^

Because molecular mechanisms underlying the pathophysiology of RE are virtually impossible to study in humans, they remain unclear for the most part. Moreover, the human VF cannot be biopsied repeatedly. Valid animal studies are scarce^10^ but also insufficient in this regard because controlled phonation is not feasible. However, in order to establish profound treatment strategies, it is mandatory to understand the underlying pathophysiology. The aim of this study was to explore the cellular mechanisms of human VF fibroblasts (hVFF) exposed to cigarette smoke extract (CSE) with or without additional vibrational stress in an experimental in vitro setting by using a phonomimetic bioreactor.

## MATERIALS AND METHODS

### Cell Culture

Immortalized hVFF (isolated from VF of a 59‐year‐old female donor) were kindly provided by Prof. S. Thibeault (University of Wisconsin‐Madison, Madison, WI^11^) and were cultivated in standard medium (SM), consisting of Dulbecco's Modified Eagle's Medium (DMEM, 4.5 g/L glucose, Sigma Aldrich, Vienna, Austria), 10% fetal bovine serum (FBS, Biowest, Nuaillé, France), and 100 μg/mL Normocin (Invivogen, San Diego, CA), as previously described.^12^

Cells were seeded in SM at a density of 15000 cells/cm^2^ in flexible BioFlex culture plates (Flexcell International, Burlington, NC) unless indicated otherwise. SM was replaced the following day with CSE or air‐bubbled control (ABC) medium, and plates were cultured for 5 days at 37°C in a humidified atmosphere with 5% CO_2_ under static or dynamic conditions, with medium change every day. Harvests for analysis were performed following a 1‐hour rest period after vibration at the end of the experiments.

ABC medium consisted of DMEM, 10 mM HEPES (Sigma Aldrich), 1% FBS (Biowest), and 100 μg/mL Normocin (Invivogen).

Generation of CSE medium was performed as described previously.^13^ Briefly, a 100 mL Erlenmeyer flask with a 5 mm polypropylene tube was guided through the flasks’ rubber cap and connected to a 200 μm borosilicate glass frit at its lower end. The lateral opening of the Erlenmeyer flask was connected to a 60 mL syringe via a silicone tube. Total extraction process consisted of three full‐strength Marlboro cigarettes (Philip Morris International Inc., New York City, NY), which were inserted into the upper end of the polypropylene tube, lit, and smoked by applying negative pressure via the syringe. For each cigarette, 6 to 7 cycles of smoking (50 mL over 20 seconds) and a 20‐second pause were applied, and the cigarette smoke was bubbled into 60 mL of ABC medium. During extraction, the medium was continuously mixed via a magnetic stir bar (7 mm length, 600 rpm). Subsequently, the resulting CSE medium was subjected to sterile filtration (0.22 μm pore size), and absorbance was measured from 200 nm to 620 nm in 10 nm steps using UV‐Star^(R)^ micro plates (Greiner Bio One, Frickenhausen, Germany). ABC medium was used to dilute the original CSE to an optical density (OD) at 240 nm of 1 (blanked against ABC medium), which was defined as 100% CSE. Further dilutions of 5% CSE, which were used for experiments, were made using ABC medium.

### Phonomimetic Bioreactor

A phonomimetic bioreactor designed by our group was used for delivering vibration to the cells.^14^ In short, hVFF were seeded in flexible‐bottomed plates and placed in a custom‐made housing containing a loudspeaker. To simulate an entire day of intensive voice use, the cells were exposed to a predefined audiostimulation protocol. This was comprised of 8 hours static, followed by 16 hours composed of 1‐minute vibration (bidirectional sinusoidal linear frequency chirp between 50 Hz and 250 Hz, chirp‐rate ± 100 Hz/s) and 1‐minute static, resulting in an effective duration of 8 hours of vibration per day. This pattern was repeated for a total duration of 5 days. After a 1‐hour rest period, cells and supernatants were harvested for subsequent analyses.

### Lactate Dehydrogenase Assay

Quantification of cytotoxicity was performed with cell culture supernatants using the Pierce LDH [lactate dehydrogenase] Cytotoxicity Assay Kit (Thermo Scientific, Waltham, MA). For each experiment, a maximum LDH activity control was run in parallel to the static conditions of interest by seeding cells at the same density in 2 wells of a 24‐well plate. 45 minutes before sampling the supernatants, 10X lysis buffer was added to these wells, followed by further incubation at 37°C and 5% CO_2_. Absorbance was measured using the Spectramax Plus 384 Microplate Reader (Molecular Devices, San Jose, CA). The LDH activity of the samples was expressed as percentage of the maximal LDH activity.

### Isolation and Analysis of RNA

RNA isolation was performed using the miRNeasy Mini Kit (Qiagen, Hilden, Germany) according to the manufacturer's instructions. Purified RNA was eluated in RNase‐free water; concentration was determined on the NanoDrop 2000c (Thermo Scientific); and cDNA synthesis was performed using the QuantiTect Reverse Transcription Kit (Qiagen) according to the manufacturer's protocol. Reverse transcription–quantitative polymerase chain reaction was performed in technical triplicates as described previously.^15^ Primer sequences are provided in Table I. C_T_ values were calculated with the AbsQuant/2nd Derivative Max method of the LightCycler 480 software (Roche, Vienna, Austria), and relative quantification of expression levels was performed using the geometric mean of beta‐2 microglobulin and ubiquitously expressed transcript protein as internal reference RNAs according to the 2^−ΔΔC^_T_ method.

**TABLE I:** Primer sequences used for RT‐qPCR.

Gene	Gene Symbol	Forward Primer	Reverse Primer
Alpha smooth muscle actin	ACTA2	CGTTACTACTGCTGAGCGTGA	GCCCATCAGGCAACTCGTAA
Beta‐2‐microglobulin	B2M	AGGCTATCCAGCGTACTCCA	CGGATGGATGAAACCCAGACA
Collagen I α1	COL1A1	CCCCGAGGCTCTGAAGGT	GCAATACCAGGAGCACCATTG
Prostaglandin‐endoperoxide synthase 2	PTGS2/COX2	AGTGCGATTGTACCCGGACAGGA	TGCACTGTGTTTGGAGTGGGTTTCA
Fibronectin 1	FN1	CTGCAAGCCCATAGCTGAGA	GAAGTGCAAGTGATGCGTCC
Hyaluronan synthase 2	HAS2	ATGCTTGACCCAGCCTCATC	TTAAAATCTGGACATCTCCCCCAA
Hyaluronan synthase 3	HAS3	ATCATGCAGAAGTGGGGAGG	GAGTCGCACACCTGGATGTA
Hyaluronidase 1	HYAL1	AGCCTAGGTTGTCCTCGACC	GCATTCCAGACGGTGGTGAA
Hyaluronidase 2	HYAL2	CGTGGTCAATGTGTCCTGGG	CCCAGGACACATTGACCACG
Matrix metalloproteinase 1	MMP1	CACGCCAGATTTGCCAAGAG	GTTGTCCCGATGATCTCCCC
Transforming growth factor beta 1	TGFB1	TACCTGAACCCGTGTTGCTC	GCTGAGGTATCGCCAGGAAT
Vascular endothelial growth factor A	VEGFA	GGCAGAATCATCACGAAGTGG	GGCACACAGGATGGCTTGA
Vascular endothelial growth factor C	VEGFC	AGGCCACGGCTTATGCAAG	ATGTTGCCAGCCTCCTTTCC
Vascular endothelial growth factor D	VEGFD	TGCTGGAACAGAAGACCACTC	ACAGACACACTCGCAACGAT
Ubiquitously expressed transcript protein	UXT	GCAGCGGGACTTGCGA	TAGCTTCCTGGAGTCGCTCA
**Gene**	**Gene Symbol**	**Company**	**Cat.‐No.**
Prostaglandin‐endoperoxide synthase 1	PTGS1/COX1	Bio‐Rad	qHsaCID0009735

RT‐qPCR = reverse transcription–quantitative polymerase chain reaction.

### Enzyme‐Linked Immunosorbent Assay

Cell culture supernatants were centrifuged (300 × g/ 10 min/4°C), decanted and hyaluronic acid (HA) was measured using the Quantikine ELISA [enzyme‐linked immunosorbent assay] Kit (R&D Systems) according to the manufacturer's protocol. Samples were prediluted 1:30 with Calibrator Diluent RD5‐18 and assayed with HA standards on the same plate. OD values at 540 nm were subtracted from OD values at 450 nm. Concentration of samples was measured using the Spectramax Plus 384 Microplate Reader (Molecular Devices).

### Magnetic Luminex^(R)^ Assay

Centrifuged cell culture supernatants (300 × g/10 min/4°C) were decanted and used to perform a Human Magnetic Luminex^(R)^ Assay (R&D Systems) with a multiplex plate measuring collagen I alpha 1 (COL1A1), matrix metalloproteinase 1 (MMP1), fibronectin 1 (FN1), hepatocyte growth factor (HGF), interleukin 8 (IL‐8), and vascular endothelial growth factor (VEGF) –A and –C according to the manufacturer's protocol on the Bio‐Plex 200 assay reader. Final concentrations were calculated using Bio‐Plex Manager Software, version 6.2. (both Biorad, Hercules, CA).

### Statistical Analysis

For statistical analysis, technical replicates were averaged, whereas biological replicates were treated as independent variables. Statistics were performed using Graph Pad Prism 7.0 software (Graph Pad, La Jolla, CA). Normal distribution of the data was assessed using the Shapiro–Wilk test. Differences of the mean were analyzed with one‐way analysis of variance and Tukey's multiple comparison test for normally distributed data, whereas Kruskal‐Wallis and Dunn's multiple comparison test were used for nonparametric data. *P* > .05 was considered to indicate a significant difference, and values are represented as means ± standard error of the mean.

## RESULTS

### Cell Viability

**Fig. 1.**
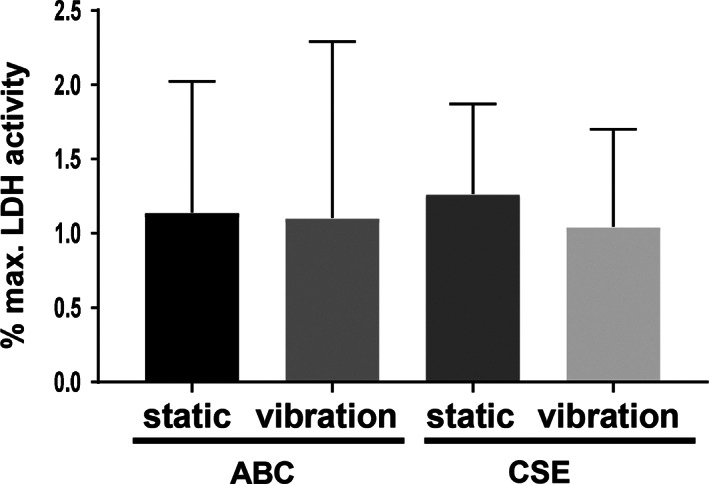
Effect of CSE exposure and/or vibration on cell viability. After 5 days of exposure to CSE and/or vibration and a 1‐hour rest period, supernatants were collected for LDH activity assay. Data are average of seven independent experiments and are represented as mean ± SEM. Statistical analysis was performed using one‐way ANOVA and Tukey multiple comparison test. ABC = air‐bubbled control; ANOVA = analysis of variance; CSE = cigarette smoke extract; LDH = lactate dehydrogenase; SEM = standard error of the mean.

To test if CSE alone or in combination with vibration alters cell viability of hVFF, LDH activity assay was performed. As shown in Figure 1, there was no difference between control cells (treated with ABC) and cells exposed either to vibration or CSE or the combination of both.

### Gene Expression and Protein Synthesis

#### Extracellular Matrix‐Related Proteins

**Fig. 2.**
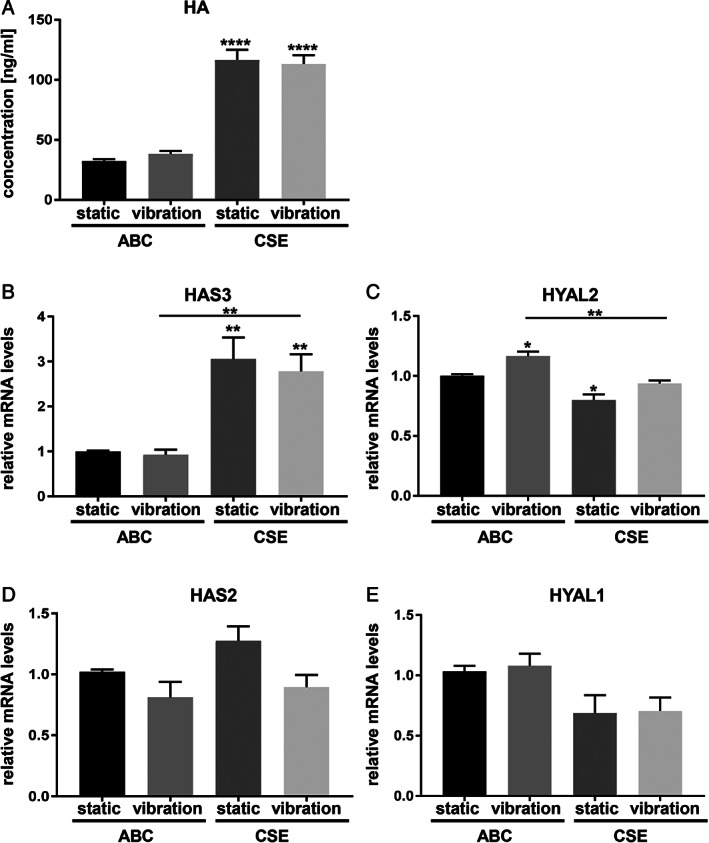
Effect of CSE exposure and/or vibration on HA synthesis and HA‐related gene expression. After 5 days of exposure to CSE and/or vibration and a 1‐hour rest period, supernatants were collected for ELISA to quantify values of HA (A). RT‐qPCR analysis of HA‐related gene expression (B‐E) was performed (normalized to B2M and UXT geometric mean, relative to ABC‐treated cells under static condition). Data are average of 6 (for ELISA) and 4 (for RT‐qPCR) independent experiments and are represented as mean ± SEM. Statistical analysis was performed using one‐way ANOVA (normally distributed data) or Kruskall‐Wallis test (nonparametric data). *P* values show significance compared to ABC‐treated cells under static condition or as indicated. * *P* < .05, ** *P* < .01, **** *P* < .0001. ABC = air‐bubbled control; ANOVA = analysis of variance; B2M = beta‐2 microglobulin; CSE = cigarette smoke extract; ELISA = enzyme‐linked immunosorbent assay; HA = hyaluronic acid; HAS2 = hyaluronan synthase 2; HAS3 = hyaluronan synthase 3; HYAL1 = hyaluronidase 1; HYAL2 = hyaluronidase 2; RT‐qPCR = reverse transcription–quantitative polymerase chain reaction; SEM = standard error of the mean; UXT = ubiquitously expressed transcript protein.

Analysis of HA by ELISA showed a significant effect of CSE leading to higher concentrations (Fig. 2A). This result was corroborated on the gene expression level by significantly increased hyaluronan synthase (HAS) 3 and significantly reduced hyaluronidase (HYAL) 2 (Fig. 2B,2C) upon CSE exposure. Gene expression of HAS2 and HYAL1 showed a similar trend but was not significantly altered by CSE (Fig. 2D,2E).

**Fig. 3.**
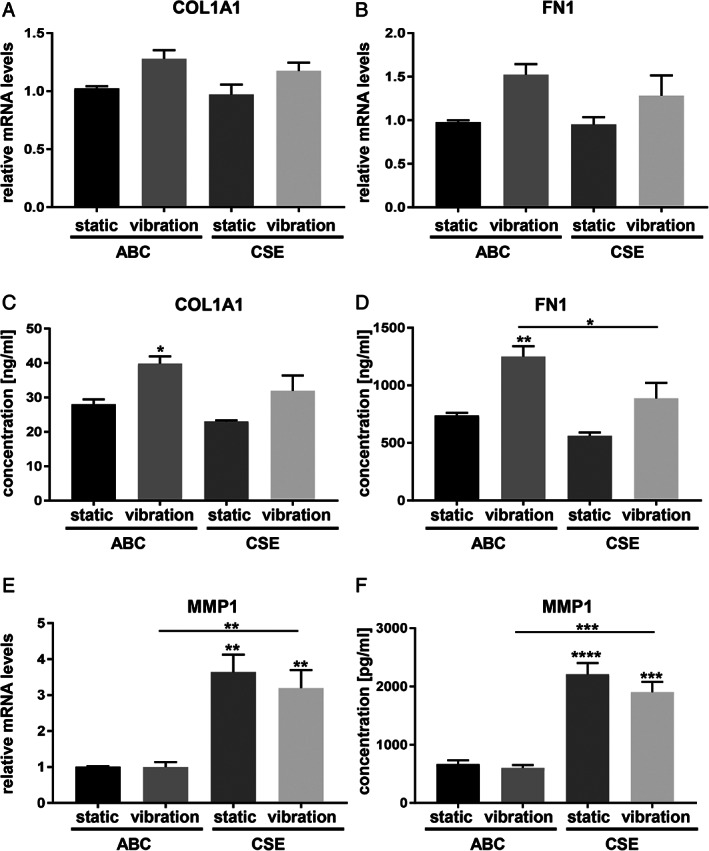
Effect of CSE exposure and/or vibration on ECM‐related protein synthesis. After 5 days of exposure to CSE and/or vibration and a 1‐hour rest period, mRNA (A, B, E) and protein levels (C, D, F) of ECM‐related protein synthesis were analyzed by RT‐qPCR and Magnetic Luminex^(R)^ assay, respectively. Data are average of four independent experiments and are represented as mean ± SEM. Statistical analysis was performed using one‐way ANOVA (normally distributed data) or Kruskall‐Wallis test (nonparametric data). *P* values show significance compared to ABC‐treated cells under static condition or as indicated. * *P* < .05, ** *P* < .01, *** *P* < .001, **** *P* < .0001. ABC = air‐bubbled control; ANOVA = analysis of variance; CSE = cigarette smoke extract; ECM = extracellular matrix; COL1A1 = collagen 1 alpha 1; FN1 = fibronectin 1; MMP1 = matrix metalloproteinase 1; mRNA = messenger RNA; RT‐qPCR = reverse transcription–quantitative polymerase chain reaction; SEM = standard error of the mean.

CSE exposure alone or in combination with vibration showed no statistically significant changes on gene expression of COL1A1 (Fig. 3A) and FN1 (Fig. 3B), although following vibration a trend toward upregulation was observed. Following vibration, COL1A1 and FN1 protein content were significantly upregulated in supernatants of ABC‐treated cells (Fig. 3C,3D). CSE had a significant effect on MMP1 gene expression and protein secretion, whereas vibration led to no additional effect (Fig. 3E,3F).

#### Fibrogenic Markers

**Fig. 4.**
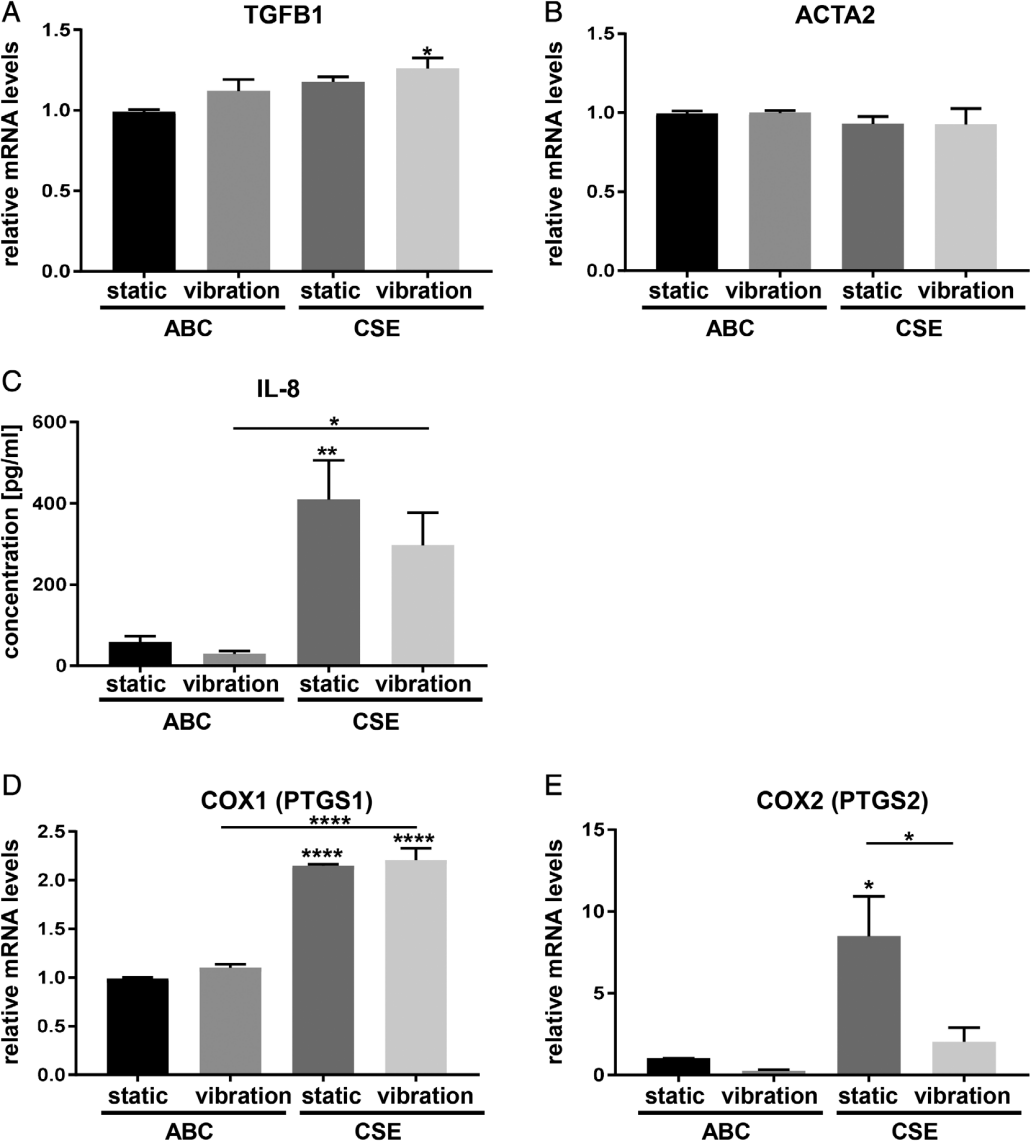
Effect of CSE exposure and/or vibration on fibrogenic and inflammatory markers. After 5 days of exposure to CSE and/or vibration and a 1‐hour rest period, mRNA (A, B, D, E) and protein levels (C) of fibrogenic and inflammatory marker genes were analyzed by RT‐qPCR and Magnetic Luminex^(R)^ assay, respectively. Data are average of four independent experiments and are represented as mean ± SEM. Statistical analysis was performed using one‐way ANOVA (normally distributed data) or Kruskall‐Wallis test (nonparametric data). *P* values show significance compared to ABC‐treated cells under static condition or as indicated. * *P* < .05, ** *P* < .01, **** *P* < .0001. ABC = air‐bubbled control; ACTA2 = alpha smooth muscle actin; ANOVA = analysis of variance; CSE = cigarette smoke extract; COX1(PTGS1) = cyclooxygenase 1; COX2(PTGS2) = cyclooxygenase 2; IL‐8 = interleukin‐8; mRNA = messenger RNA; RT‐qPCR = reverse transcription–quantitative polymerase chain reaction; SEM = standard error of the mean; TGFB1 = transforming growth factor beta 1.

Gene expression of transforming growth factor beta 1 (TGFB1) was significantly upregulated only when CSE and vibration were combined (Fig. 4A). Gene expression profiles of alpha smooth muscle actin (ACTA2) remained unchanged (Fig. 4B).

IL‐8 protein levels were significantly increased by CSE stimulation, whereas additional vibration had a diminishing effect (Fig. 4C). No changes were detected for gene expression of HGF and nuclear respiratory factor 2 (data not shown).

#### Inflammatory Markers

Cyclooxygenase (COX, also known as *prostaglandin‐endoperoxide synthase*) 1 gene expression was significantly increased after CSE exposure under static and dynamic conditions (Fig. 4). Under static conditions, CSE exposure also upregulated COX2 gene expression, which was ablated by vibration (Fig. 4E).

#### Angiogenic Factors

**Fig. 5.**
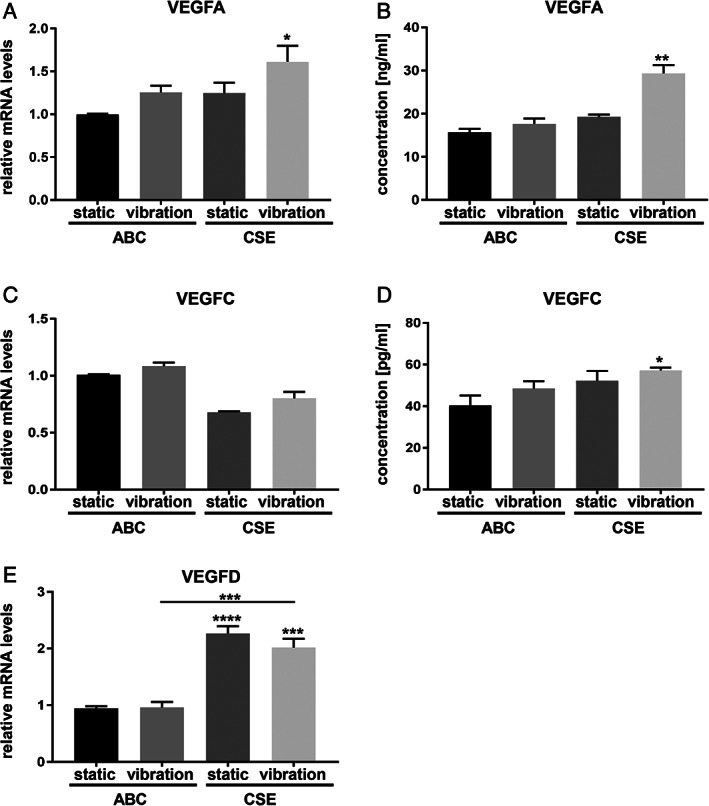
Effect of CSE exposure and/or vibration on angiogenic factors. After 5 days of exposure to CSE and/or vibration and a 1‐hour rest period, mRNA (A, C, E) and protein levels (B, D) of angiogenic factors were analyzed by RT‐qPCR and Magnetic Luminex^(R)^ assay, respectively. Data are average of four independent experiments and are represented as mean ± SEM. Statistical analysis was performed using one‐way ANOVA (normally distributed data) or Kruskall‐Wallis test (nonparametric data), *P* values show significance compared to ABC‐treated cells under static condition or as indicated. * *P* < .05, ** *P* < .01, *** *P* < .001, **** *P* < .0001. ABC = air‐bubbled control; ANOVA = analysis of variance; CSE = cigarette smoke extract; mRNA = messenger RNA; RT‐qPCR = reverse transcription–quantitative polymerase chain reaction; SEM = standard error of the mean; VEGFA,‐C,‐D = vascular endothelial growth factor A, ‐C, ‐D.

Only a combination of CSE and vibration led to a significant upregulation of VEGFA gene expression and protein level (Fig. 5A,5B) and VEGFC protein level alone (Fig. 5C,5D). CSE alone significantly stimulated VEGFD gene expression, whereas vibration showed no additional effect (Fig. 5E).

## DISCUSSION

The pathophysiology of RE is very likely multifactorial; however, despite a lot of studies in this field, the details remain unclear. Consequently, existing treatment modalities remain only symptomatic. Whereas smoking is a generally accepted risk factor, other factors (e.g., voice abuse, hormonal changes)—alone or in combination—remain unclear because these factors cannot be followed on a cellular level in humans for the reasons mentioned above. A recently developed phonomimetic bioreactor allowed us to explore the exposure to cigarette smoke and/or vibration in hVFF, a cell type that plays a central role in the development of RE.^16^, ^17^, ^18^

HA is a main component of the VF lamina propria responsible for viscoelasticity; thus, it is central for undisturbed vibration. Although vibration had no effect, we found a significant upregulation of HA concentrations after CSE exposure. This might be a protective reaction of hVFF and corroborates previous data from our group.^13^ In a clinical study, Woo described that the application of HYAL into RE diminished VF mass, highlighting the role of HA in RE.^19^ COL1A1 and FN1 are important extracellular matrix components and provide structural strength to tissue. Also, they are crucial in resisting stress and deformation. Vibration alone led to an upregulation of COL1A1 in supernatant of treated cells, with no additional effects of CSE. However, vibration‐induced protein levels of FN1 were reduced in the presence of CSE. This is in line with the histology of RE, in which a decrease of FN was found in the lamina propria.^20^ MMP1 is an interstitial collagenase involved in the turnover of collagen types I and III and is usually stable in normal resting tissues.^21^ Our results showed that the exposure to CSE lead to a significant upregulation of MMP1, reflecting an active process of tissue repair. Similar effects of CSE on MMP1 were previously shown in periodontal ligament fibroblasts,^22^ in rheumatoid arthritis synovial fibroblasts,^23^ and in lung epithelial cells.^24^, ^25^, ^26^

The inflammatory cytokine TGFB1 was significantly upregulated only when CSE exposure was accompanied by vibration. However, ACTA2, a marker of myofibroblasts, did not change, thus indicating no shift to a profibrotic phenotype of hVFF cells in our experiments. CSE induced IL‐8 production in hVFF. This is in line with previous studies in which cigarette smoke induced IL‐8 production in human bronchial epithelial cells^27^ and macrophages.^28^ IL‐8, a well‐known proangiogenic cytokine, likely plays a role in the vascular phenotype of RE.^29^

COX1 and 2, major facilitators of prostanoid‐mediated tissue inflammation, were significantly upregulated after CSE exposure. Interestingly, COX2 induction was considerably reduced when vibration was applied. This is in line with a previous study by Zhang et al., who investigated the effect of cyclic tensile strain on cigarette smoke condensate‐exposed VFF.^30^ Moreover, COX2 levels were lower in VF tissue samples of RE compared to VF carcinoma. A personality questionnaire survey of patients showed that smokers who were more extroverted and talkative tended to develop RE, whereas introverted smokers were more likely to develop VF carcinoma.^30^ Because COX2 overexpression is associated with tumor progression,^31^ downregulation of COX2 by vibration could explain why, despite chronic exposure to cigarette smoke, RE patients rarely develop malignant transformation of the VF tissue.

Laryngoscopic examination of RE VF typically shows elongated and dilated vessels. Based on electron microscopic examinations, we know that vessels in RE are often fenestrated and leaky, an effect that was linked to elevated expression of proangiogenic proteins such as VEGF. According to Sato et al., this growth factor may enhance capillary permeability and form fragile vessels.^9^ The cytoplasm of interstitial cells and inflammatory cells infiltrating the lamina propria of vessels of RE samples stained positively for VEGF. In line with these data, we found the combination of the two conditions thought to be crucial for the pathogenesis of RE—CSE and mechanical stress—to significantly increase VEGFA and VEGFC secretion from hVFF.

Although the purpose of this study was restricted to reveal the effects of vibration on the cellular level; we could not simulate all physiologically relevant in vivo forces on VF tissue in our model system. There are plenty of other aspects that need to be considered in the future. These comprise the introduction of other cell types (epithelial cells, endothelial cells, macrophages), microhistology (leaking vessels), hormonal influences, and further biomechanical features on the level of the entire VF mucosa. To date, there is no experimental setup that can cover all these requirements.

## CONCLUSION

The use of a phonomimetic bioreactor allowed us to explore the cellular effects of CSE and vibration, alone or in combination, on hVFF. CSE is considered a secured risk factor in the development of RE. The role of additional vibration remained unclear. Whereas CSE exposure alone lead to a significant upregulation of HA and MMP1, we discovered settings in which only the combination of both factors led to altered expressions such as the proinflammatory cytokine TGFB1, as well as the proangiogenic proteins VEGFA and VEGFC. At the same time, elevated levels of COX2 triggered by CSE were significantly reduced when vibration was applied. Our experimental setting can be used as an innovative in vitro RE model that will allow the study of other factors, which are possibly linked to (RE) as well as potential therapies.
